# Malignant Deciduoid Mesothelioma: case presentation of an exceptional variant and review of the literature

**DOI:** 10.1186/s12907-017-0051-2

**Published:** 2017-08-18

**Authors:** Mouna Khmou, Soumiya Echcharif, Rachad Kabbaj, Basma El Khannoussi

**Affiliations:** 1 Department of Pathology, National Institute of Oncology, Rabat, Morocco; 2 Faculty of Medicine and Pharmacy Rabat, University Mohammed V, Rabat, Morocco

**Keywords:** Mesothelioma, Deciduoid, Peritoneum, Immunohistochemistry

## Abstract

**Background:**

Malignant Deciduoid Mesothelioma (MDM) is an extremely rare variant of epithelioid mesothelioma. It was first described in young females, in the peritoneum, and its relation with asbestos was not well defined.

Later reports, have shown that this variant may also occur in the pleura, the pericardium and the tunica vaginalis of elderly people, who had been exposed to asbestos.

**Case presentation:**

We report a case of malignant deciduoid mesothelioma that occurred in the peritoneal cavity, and the omentum of a 35-year-old woman. The patient had never been exposed to asbestos.

**Conclusions:**

Through this observation, we describe clinical, histopathological, and immunohistochemical findings of deciduoid mesothelioma, and review the literature reports.

## Background

MDM is an extremely rare subtype of malignant epithelioid mesothelioma, with a poor prognosis. It was first described by Talerman et al. [1] in 1985 and Nascimento et al. [2] in 1994, characterized by the presence of cytomorphological features resembling decidual reaction [3].

Few cases have been reported in the literature to date, in the form of single case reports. A total of 25 cases of deciduoid peritoneal mesothelioma have been documented in the English literature, since this entity was first described in 1985 [1, 2, 4–17].

The aim of the present report is to describe MDM of the peritoneum, to discuss the differential diagnosis and management of this extremely rare tumour.

## Case presentation

**Fig. 1:**
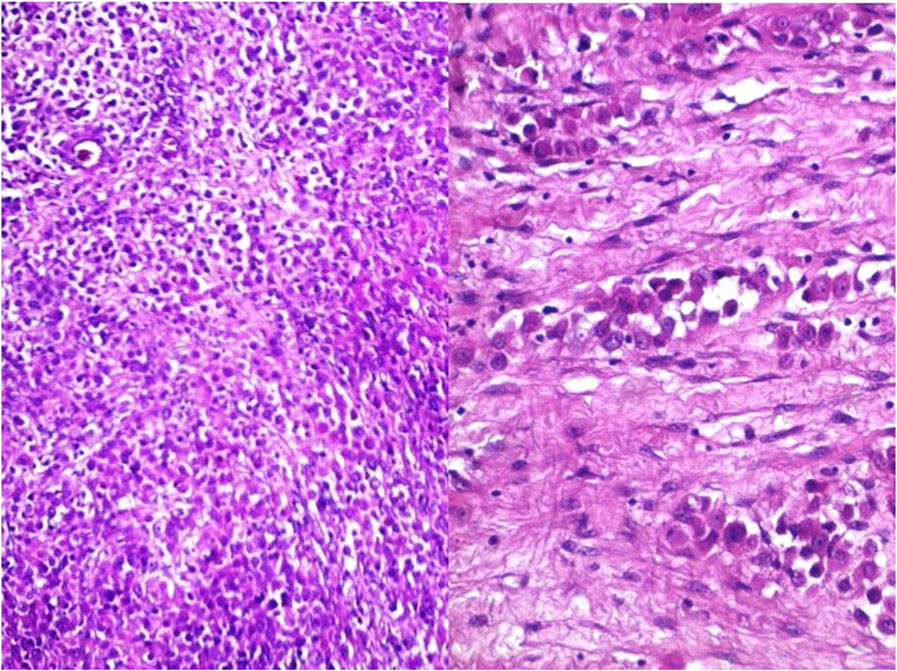
Histological section showing large solid sheets of malignant epithelioid cells

**Fig. 2:**
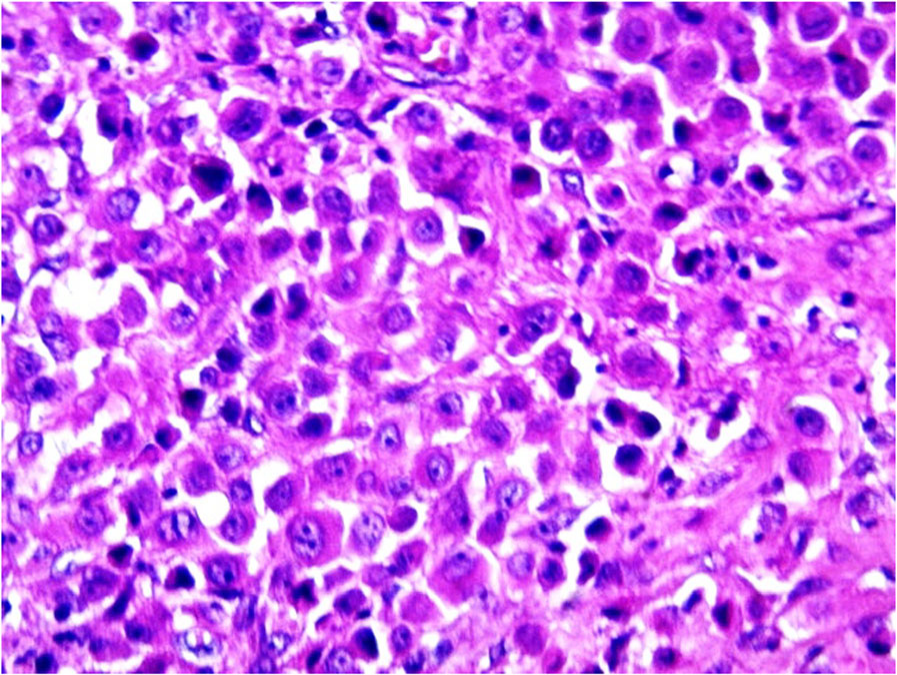
Decidual tumor cells with large eosinophilic cytoplasm and prominent nucleoli

A 35-year-old female patient, with no medical history, was admitted for an appendicular syndrome. There was no history of asbestos exposure. On physical examination, she had a low-grade fever. A generalized abdominal tenderness and distension were also reported. Abdominal ultrasound examination showed moderate ascites, and hyperechogenic mesenteric fat. An exploratory laparotomy revealed multiple firm nodules, white to gray, infiltrating the peritoneal cavity, and the omentum. A biopsy was performed. We received four firm, gray white fragments. Microscopically, H&E stain showed sheets of malignant cells, formed exclusively by large pleomorphic epithelioid cells with abundant, eosinophilic cytoplasm. The nuclei were large and vesicular, with prominent nucleoli (Figs. 1 and 2). The cell borders were variably well defined. Focally tumor cells showed finely vacuolated cytoplasm, with binucleated and multinucleated cells. Areas of necrosis were commonly seen. Lymphoplasmacytic and neutrophilic infiltration was moderate to dense.

**Fig. 3:**
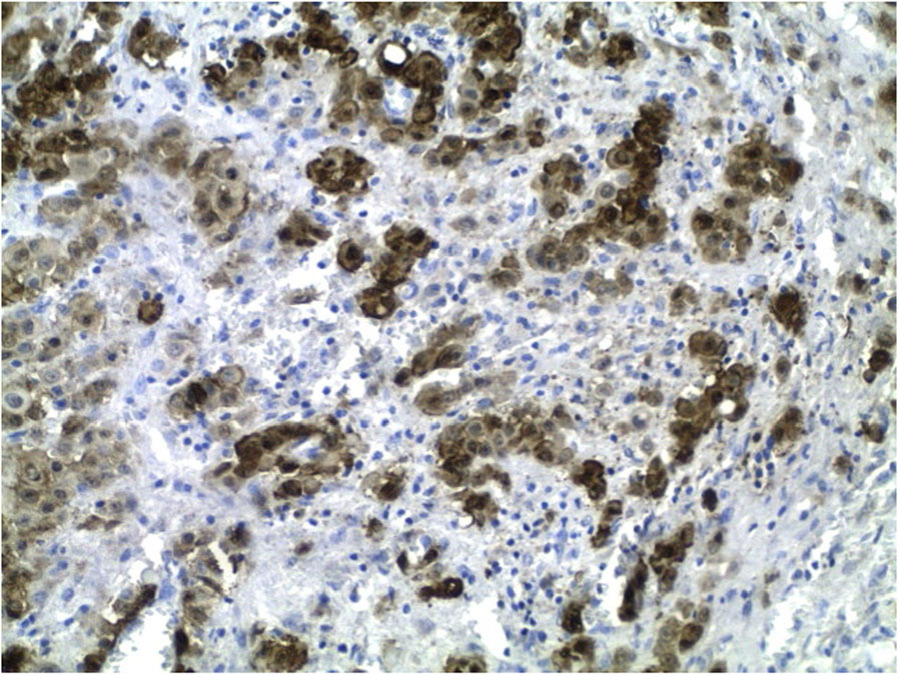
tumor cells with positive immunoreactivity to calretinin

In immunohistochemistry, tumor cells were positive for cytokeratin AE1/AE3, calretinin (Fig. 3), D2–40, CK5/6 and WT-1. They were negative for Ber-EP4, CEA, estrogen (ER) and progesterone (PR) receptors, and HMB-45. The microscopic and immunohistochemical findings were compatible with deciduoid malignant mesothelioma. The patient received 5 cycles of chemotherapy. She presently has a survival of 12 months.

## Discussion

Deciduoid mesothelioma was initially reported in the peritoneum of young women, with no history of asbestos exposure. Later reports have demonstrated that this entity could occur in the pleura and pericardium of older men and women who had been exposed to asbestos [15, 16].

This designation was first introduced by Nascimento et al. [2] in 1994, to describe a rare variant of epithelioid mesothelioma, that reflect a morphological resemblance with decidua or decidual type changes [15].

The scarcity of this entity was highlighted by Ordonez and al. [15] in the latest review of malignant deciduoid mesothelioma. Only 25 cases of deciduoid peritoneal mesothelioma have been reported until today [1, 2, 4–17]. Seventeen of these patients were female, with ages ranging from 8 to 78 years. Nineteen cases were unrelated etiologically to asbestos. The five cases associated with asbestos exposure were noted in elderly patients (Table 1).

**Table 1:** Summary of the cases of Malignant Deciduoid Mesothelioma in the peritoneum, reported in the literature

Author(s)	Sex/age	Asbestos exposure	Type of specimen	Histology
Talerman et al. [1]	F/13	No	Biopsy	Deciduoid
Nacimento et al. [2]	F/23	No	Biopsy	Deciduoid
	F/24	No	Biopsy	Deciduoid
Orosz et al. [4]	F/15	No	Biopsy	Deciduoid
Shanks et al. [5]	F/53	Yes	Biopsy	Deciduoid
	M/65	Yes	Biopsy + autopsy	Deciduoid
	M/55	Yes	Tumor resection	Deciduoid + tubular and sarcomatoid areas
	F/55	No	Biospy	Predominantly tubulopapillary + sarcomatoid areas, deciduoid cells
Desai et al. [6]	F/53	NA	Tumor resection	Deciduoid
Gillespie et al. [7]	F/50	No	Peritoneal cytology	Deciduoid
Okonkwo et al. [8]	F/30	No	Tumor resection	Deciduoid
Shia et al. [9]	F/51	NA	Tumor resection	Deciduoid + tubulopapillary
	M/56	Yes	Resected specimen	Deciduoid + tubulopapillary
Chung et al. [10]	F/47	No	Resected tumor	Deciduoid
Maeda et al. [11]	F/24	No	Resected tumor	Deciduoid
Kimura et al. [12]	M/70	No	Peritoneal biopsy,	Deciduoid
			autopsy	
Mourra et al. [13]	F/41	No	Tumor resection	Deciduoid with pleomorphic nuclei
Ustun et al. [14]	F/59	No	Tumor resection	Deciduoid
Ordonez [15]	M/78	No	Biopsy	Deciduoid
	M/62	No	Peritoneal nodules	Deciduoid
	M/56	No	Biopsy	Deciduoid
	F/39	No	Biopsy	Deciduoid
Huang [16]	M/18	No	Biopsy	Deciduoid
	F/ 64	Yes	Biopsy	Deciduoid
Wolff-bar [17]	F/8	No	Cytoreductive surgery	Deciduoid + tubulopapillary
Our case	F/35	No	Biopsy	Deciduoid

*NA* Not Available

Clinically, symptoms are often insidious and nonspecific, causing a delay in diagnosis; the imaging findings are also not specific. Histologically, there are four major subtypes of mesothelioma: epithelioid, sarcomatoid, desmoplastic and biphasic. Deciduoide mesothelioma is a rare subtype of epithelioid mesothelioma, in which a wide range of histopathological patterns is described [18, 19].

Microscopically, deciduoid mesothelioma is composed of large, polygonal, or round cells, arranged in solid nests and in trabecula [13, 18, 19]. The cytoplasm is abundant eosinophilic with well-defined cell borders [18, 19]. The nuclei is large and vesicular, with prominent nucleoli. Nuclear pleomorphism, nuclear pseudoinclusions, multinucleated cells have been reported. Mitotic index is variable, with high mitotic activity and atypical mitoses observed in some cases. Several features described in the literature include the presence of foamy cells, with clear cytoplasm, and mucinous or myxoid stroma [15].

A large immunohistochemical panel is essential for accurate diagnosis, and help in distinguishing differentiel diagnosis. The International Mesothelioma Panel and the WHO recommend to use at least 2 mesothelial and 2 carcinoma markers, in addition to a pancytokeratin [19, 20]. The choice of immunohistochemical markers used varies depending on the histologic type of mesothelioma, the location of the tumor (pleural versus peritoneal), and the type of tumor being considered in the differential diagnosis [20].

Based on their specificity and sensitivity, calretinin, CK5/6, EMA, WT-1 and D2–40 are de the best mesothelial markers, and are positive in MDM [19]. Ber-EP4, B72.3, and carcinoembryonic antigen are not expressed in mesothelioma. They are the most commonly used markers to eliminate carcinoma (primary peritoneal carcinoma, and secondary peritoneal carcinomatosis) [19]. Others markers can be added to assist in the differential diagnosis.

Our findings illustrated that calretinin, AE1/AE3, CK5/6, D2–40 and WT-1 were positive. Ber-EP4, CEA showed a negative result, respectively, which is consistent with the literature.

Peritoneal Deciduoid mesotheliomas of women should be distinguished from diffuse pseudotumoral deciduosis. This condition has been frequently associated with pregnancy, but also observed in the perimenarchal and postmenopausal periods [15]. Microscopically, the nuclei are smaller and have dark dense chromatin [13]. Decidual cells lack expression of pancytokeratin and calretinin. However, they strongly express CD10, alpha inhibin, estrogen and progesterone receptors [15, 21].

Trophoblastic neoplasia is also important to consider in the differential diagnosis [21]. The neoplastic cells in this condition express HCG [15].

Positive staining for AE1/AE3 and calretinin are useful to exclude Rhabdomyosarcoma, epithelioid gastrointestinal stromal tumor (GIST), melanoma and epithelioid angiosarcoma [16, 21]. Immunohistochemical staining should easily distinguish these entities, using Desmin and myogenin for rhabdomyosarcoma; C-kit and Dog1 for GIST; Melan-A, HMB-45 for melanoma; and ERG for angiosarcoma [21].

Chemotherapy and cytoreductive surgery are usually the best therapeutic options. Cisplatin and pemetrexed have demonstrated a significant survival advantage, and are considered as firstline treatment [16, 17].

The deciduoid subtype was regarded by some authors to be a more aggressive variant [6]. However, some recent reviews of reported cases did not reveal a significantly worse survival in patients with deciduoid features. There are no large survival studies for MDM due to its rarity [9].

## Conclusions

In summary, through this observation, we want to highlight the rarity of this variant. It is important for pathologists to recognize this morphological variant of epithelioid mesothelioma, easily confused with other diangnosis, and discuss the available treatment options. We believe that more studies on a larger number of cases may be helpful in further defining this entity.

## Abbreviations

ER: estrogen receptors
H&E: Hematoxylin and eosin
MDM: Malignant Deciduoid Mesothelioma
PR: progesterone receptors
WHO: World Health Organization
